# Job Strain, Burnout, and Suicidal Ideation in Tenured University Hospital Faculty Staff in France in 2021

**DOI:** 10.1001/jamanetworkopen.2023.3652

**Published:** 2023-03-28

**Authors:** Martin Dres, Marie-Christine Copin, Alain Cariou, Muriel Mathonnet, Raphael Gaillard, Tait Shanafelt, Bruno Riou, Michael Darmon, Elie Azoulay

**Affiliations:** 1 Sorbonne Université, INSERM, UMRS 1158, Paris, France; 2 Department of Critical Care Medicine, Pitié-Salpêtrière Hospital, Assistance Publique-Hôpitaux de Paris, Paris, France; 3 Université Angers, CHU Angers, INSERM, CNRS, CRCI2NA, Angers, France; 4 Paris Cité University, Paris, France; 5 Department of Critical Care Medicine, Cochin Hospital, Assistance Publique-Hôpitaux de Paris, Paris, France; 6 Department of Digestive, General, and Endocrinology Surgery, University of Limoges, INSERM, UMR 1308, Limoges, France; 7 Paris Cité University and Sorbonne Université, INSERM, UMR S894, Paris, France; 8 Centre Hospitalier Sainte-Anne, Paris, France; 9 Centre de Psychiatrie et Neurosciences, Paris, France; 10 Stanford School of Medicine, Stanford, California; 11 Sorbonne Université, INSERM, UMR 1166, Fondation pour l’Innovation en Cardiométabolisme et Nutrition, Paris, France; 12 Emergency Department, Pitié-Salpêtrière Hospital, Assistance Publique-Hôpitaux de Paris, Paris, France; 13 Department of Critical Care Medicine, Saint Louis Hospital, Assistance Publique-Hôpitaux de Paris, Paris, France

## Abstract

**Importance:**

The ability to attract and retain university hospital faculty staff is in jeopardy because of the high levels of mental symptoms in this professional group.

**Objective:**

To examine the prevalence and determinants of symptoms of severe burnout, job strain, and suicidal ideation in tenured associate and full professors in university hospitals.

**Design, Setting, and Participants:**

This nationwide cross-sectional study offered online surveys to 5332 tenured university hospital faculty members in France from October 25, 2021, to December 20, 2021.

**Exposures:**

Burnout and job strain.

**Main Outcomes and Measures:**

Participants completed the 22-item Maslach Burnout Inventory and a 12-item job strain assessment tool, reported suicidal ideation, and used visual analog scales to evaluate unidimensional parameters. The primary outcome was presence of severe burnout symptoms. Factors associated with mental health symptoms were identified by multivariable logistic regression.

**Results:**

Completed questionnaires were returned by 2390 of 5332 faculty members (response rate, 45%; range, 43%-46%). Tenured associate professors were a median of 40 (IQR, 37-45) years old with a sex ratio of 1:1, whereas tenured full professors were a median of 53 (IQR, 46-60) years old with a sex ratio of 1:5. Of 2390 respondents, 952 (40%) reported symptoms of severe burnout. Symptoms of job strain (296 professors [12%]) and suicidal ideation (343 professors [14%]) were also reported. Compared with full professors, significantly more associate professors reported feeling overwhelmed at work (496 [73%] vs 972 [57%]; *P* < .001), considering resignation (365 [54%] vs 834 [49%]; *P* = .004), or considering a career change (277 [41%] vs 496 [29%]; *P* < .001). Factors independently associated with less burnout were a longer time being a professor (adjusted odds ratio [aOR], 0.97; 95% CI, 0.96-0.98 per year of age), sleeping well (aOR, 0.88; 95% CI, 0.83-0.92), feeling valued by colleagues (aOR, 0.91; 95% CI, 0.86-0.95 per visual analog scale point) or the public (aOR, 0.92; 95% CI, 0.88-0.96 per visual analog scale point), and accepting more tasks (aOR, 0.82; 95% CI, 0.72-0.93). Factors independently associated with more burnout were having a nonclinical position (OR, 2.48; 95% CI, 1.96-3.16), reporting work encroachment on private life (OR, 1.17; 95% CI, 1.10-1.25), feeling the need to constantly put on a brave face (OR, 1.82; 95% CI, 1.32-2.52), considering a career change (OR, 1.53; 95% CI, 1.22-1.92), and having experienced harassment (OR, 1.52; 95% CI, 1.22-1.88).

**Conclusions and Relevance:**

These findings suggest that the psychological burden on tenured university hospital faculty staff in France is considerable. Hospital administrators and health care authorities should urgently develop strategies for burden prevention and alleviation and for attraction of the next generation.

## Key Points

**Question:**

How much burnout, job strain, and suicidal ideation are experienced by tenured university hospital faculty staff in France?

**Findings:**

In a nationwide cross-sectional survey of 2390 tenured university hospital faculty members, 40% of participants reported severe burnout, 12% reported job strain, and 15% reported suicidal ideation. Risk factors amenable to improvement included work encroachment on private life and perceived lack of support from the institution.

**Meaning:**

These findings underscore the urgent need for measures to improve working conditions for university hospital faculty and increase job attractiveness for the next generation.

## Teaser

This cross-sectional study assesses severe burnout and job strain in tenured faculty members at university hospitals in France.

## Introduction

University hospitals can be successful only if they are composed of committed, enthusiastic, and motivated faculty.^1^ Tenure is obtained through a challenging and lengthy selection process for which medical expertise, teaching, research, and management skills are required. The drive and productiveness of faculty depend on personality, self-efficacy, and work-related factors, including workload, autonomy, job control, and support.^2^

The extent to which faculty feel that their needs are met has received little attention. Pressure on faculty is mounting. The demand for care is rising, and administrative work is expanding. The increasing number of trainees and the greater attention to assessing the acquisition of specific competencies add to the workload.^3^ Funding is a matter of constant concern in research. Faculty members are engaged in an endless battle to provide the requested quantity while maintaining optimal quality.^4^ Their work is rewarding but also involves huge responsibilities and creates massive stress.^5^ All of these commitments create a work-life imbalance that is likely to worsen over time. Moreover, excessive rivalry is common, notably across generations. The increasing use of productivity metrics can pit faculty members against one another.^6^ High levels of professional stress, mental illness, substance use, and burnout have been reported among university hospital faculty.^2,7,8,9^

Nonetheless, data on mental health symptoms in tenured university hospital faculty are scarce. In the US, burnout has been reported in one-third of physicians^2,4,10,11^ and faculty at a large university hospital.^12^ One-third of faculty working at a large academic center were also at high risk for suicide and depression.^13^ Substantial sex differences were reported.^14^ Mental health symptoms were associated with resigning,^15^ notably in the youngest faculty.^16^ The number of candidates to faculty positions in university hospitals is decreasing. Understanding the determinants of mental health distress in tenured faculty is crucial to ensure that patients receive high-quality and compassionate care and that faculty are appropriately supported in research and teaching.

The primary objective of this nationwide cross-sectional survey was to examine the prevalence and risk factors of symptoms of severe burnout in tenured associate and full professors working in university hospitals in France. The secondary objectives were to assess the prevalence and risk factors of job strain and suicidal ideation.

## Methods

The survey was approved by the Société de Réanimation de Langue Française Ethics Committee in July 2021. This report complies with the Checklist for Reporting of Survey Studies.^17^ This cross-sectional study followed the Strengthening the Reporting of Observational Studies in Epidemiology (STROBE) reporting guideline.

### Survey Instrument

The instrument was developed by experts in survey design, psychology, and medical faculty issues. It was then tested twice in succession by a panel of tenured university hospital faculty members and revised according to the results. The final instrument was posted on a secure web-based application specifically designed for developing surveys and managing the resulting database. The participants completed the survey anonymously.

The survey questions covered 7 domains: personal characteristics and professional experience; organization of work time, including for patient care, research, teaching, and administrative tasks; symptoms of job strain and job demand; career advancement and perspectives; symptoms of burnout; and personal feelings. Symptoms of burnout were measured using the validated French-language version of the 22-item Maslach Burnout Inventory (Human Services version),^18^ which includes 3 subscales: emotional exhaustion (9 items), depersonalization (5 items), and personal accomplishment (8 items). Each item is scored from 0 (never) to 6 (every day). Respondents with high emotional exhaustion (score, ≥27) and/or high depersonalization (score, ≥10) scores were considered to have symptoms of burnout.^19^ To assess job strain, the survey had a 12-item scale derived from the Job Content Questionnaire,^20^ with 3 domains: job demand, job control, and outside-the-job support.^21^ The job strain score was computed by subtracting the demand subscore from the sum of the control and social support subscores; thus, the job strain score was lower when the job demand subscore was lower and/or the social support and job control subscores were higher. The participants also completed several visual analog scales (VASs) to evaluate unidimensional parameters, with 0 indicating best possible and 10 worst possible. Suicidal ideation was assessed. Participants were asked about any professional changes they were considering for the next 3 years, such as a career change, resignation, sabbatical, switching to private practice only, moving to another city or country, or early retirement.

### Population

All tenured full professors and associate professors in French university hospitals were sent an email invitation to complete an online survey. The survey link was left open from October 25, 2021, to December 20, 2021. Three reminders were sent by email to nonrespondents. Faculty representatives and medical school deans worked to inform faculty about the survey and to encourage participation. Presence of symptoms of severe burnout was the primary outcome. Job strain, defined as a Job Content Questionnaire score of less than −2, was among the secondary outcomes, together with suicidal ideation and other binary variables evaluated in the survey.

### Statistical Analysis

Analyses were performed by an independent statistician (M. Dres). Data are reported as median (IQR) or number (percentage). No imputation was performed for partial or incomplete answers. The proportion of missing data was 2.6% overall and 0.4% for data on study outcomes. Severe burnout syndrome, job strain, and suicidal ideation were handled as binary variables.

To identify variables independently associated with each outcome, we built logistic regression models. Variable selection was by conditional stepwise regression with critical *P* values of .20 for entry into the model and .10 for removal. Five variables considered downstream to the primary and secondary outcomes were not included in these models, namely, feeling frustrated with work, regretting the career choice, considering a career change, daily alcohol use, and daily psychotropic drug use. Interactions and correlations between explanatory variables were checked carefully. Continuous variables for which log linearity was not confirmed were transformed into categorical variables based on the median or IQR. Calibration, discrimination, and relevancy of the final models were assessed. Residuals were plotted and the distributions inspected. Adjusted odds ratios (aORs) of variables present in the final model are reported with their 95% CIs.

Three sensitivity analyses were preplanned to assess the robustness of the findings: men vs overall population, full professor vs overall population, and nonsurgeons vs overall population. These sensitivity analyses were to be performed if the 3 variables (male, full professor, and nonsurgeon) were not selected for the model.

The statistical analyses were performed using R software, version 3.6.2 (R Foundation for Statistical Computing). All analyses were 2-tailed, and *P* < .05 was considered statistically significant.

## Results

### Participants

A total of 2390 participants of the 5332 tenured university hospital faculty members in France during the study period (1260 associate and 4072 full professors) completed the survey (response rate, 45%; range, 43%-46%). Tenured associate professors had a median (IQR) age of 40 (37-45) years with a sex ratio of 1:1, whereas tenured full professors had a median (IQR) age of 53 (46-60) years with a sex ratio of 1:5. Table 1 reports the main characteristics of the participants. The male-to-female ratio was 7:3 (1574 [66%] men and 802 [34%] women), but 45% of associate professors and 50% of participants in the lowest age quartile were female. The most time-consuming activities were patient care (40% of the work time) and research and teaching (30%), but administrative tasks accounted for 20% and transversal activities for 10% of the work time.

**Table 1.** Main Characteristics of the 2390 Survey Participants

Characteristic	No. of missing values	Tenured professors, No. (%)	
		Associate (n = 677)	Full (n = 1699)
Age, median (IQR), y	185	40 (37-45)	53 (46-60)
Sex	51		
Male		341 (50)	1233 (78)
Female		336 (50)	466 (22)
Specialty	0		
Medical	0	260 (38)	861 (51)
Surgical	0	79 (12)	362 (21)
Nonmedical, nonsurgical	0	282 (42)	398 (23)
Other	0	56 (8)	78 (5)
Daily psychotropic drug use	14	70 (10)	163 (10)
Chronic disease	28	83 (12)	289 (17)
Department head	0	177 (26)	1248 (73)
Research laboratory head	0	75 (11)	534 (31)
No. of publications in last 3 y, median (IQR)	348	7 (4-10)	12 (7-15)
No. of students mentored in last 3 y, median (IQR)	14	6 (3-9)	9 (6-12)
Hours worked per week (without hours worked from home), median (IQR)	37	60 (53-70)	65 (60-73)
No. of weekends at work per 3 mo, median (IQR)	71	3 (0-5)	2 (0-4)
No. of nights at work per month, median (IQR)	103	2 (0-10)	0 (0-6)
Days off always on desired dates	14	537 (79)	1379 (82)
Also works in private practice	31	33 (5)	339 (20)
No. of half-days at conferences per year, median (IQR)	58	10 (5-12)	12 (8-20)
Perceived need to constantly put on a brave face	27	574 (85)	1471 (86)
Suicide attempt by a colleague	27	123 (18)	413 (24)
Has experienced psychological harassment	32	277 (41)	723 (42)
Feel they have to fill in for others	33	328 (48)	686 (40)
Work time spent, median (IQR), %			
On organization	80	10 (5-20)	20 (10-30)
On research and teaching	168	40 (20-54)	35 (20-70)

The median (IQR) VAS scores for quality of relationships were very high for colleagues (8 of 10 [7-9]) and head nurses (8 of 10 [6-9]), high for deans (7 of 10 [6-9]), and only fair for medical directors (5 of 10 [4-7]) and administrative directors (5 [5-7]). As shown in Table 2, compared with full professors, associate professors gave lower ratings for work climate and feeling valued at work; they reported more often that their career fell short of their expectations and that they regretted choosing it. The activity scored as most rewarding was patient care (median [IQR] score, 70 of 100 [50-80]), followed by teaching (60 of 100 [50-80]) and research (50 of 100 [20-70]). Administrative tasks were given a low score (median [IQR], 20 of 100 [10-50]). Shortage of resources was reported by 2031 participants (85%) for research activity, 1960 (82%) for administrative tasks, 1721 (72%) for patient care, and 1434 (60%) for teaching. More associate professors were considering resigning (365 [54%] vs 834 [49%]; *P* = .004) or switching to another career (277 [41%] vs 496 [29%]; *P* < .001), and 496 (73%) of them felt overwhelmed at work compared with 972 (57%) of full professors (*P* < .001). However, 1 in 4 full professors (n = 466) were considering early retirement.

**Table 2.** Challenges and Symptoms Reported by the 2390 Survey Participants

Challenge or symptom	No. of missing values	Tenured professors, median (IQR)^a^	
		Associate (n = 677)	Full (n = 1699)
Climate at work	25	7 (5 to 8)	8 (7 to 9)
Research funding easily obtained	60	3 (1 to 5)	3 (2 to 5)
Feels valued by colleagues	32	7 (5 to 8)	8 (6 to 8)
Feels valued outside the hospital	32	8 (7 to 9)	8 (8 to 9)
Work encroaches on private life	32	8 (7 to 9)	8 (7 to 10)
Career is unfolding as expected	31	5 (3 to 7)	6 (5 to 8)
Regrets the career choice	30	5 (2 to 6)	2 (1 to 5)
Sufficient resources, No. (%)			
For research	54	70 (10)	188 (11)
For patient care	59	157 (23)	421 (25)
Plans being considered for the next 3 y, No. (%)			
Sabbatical	28	454 (67)	1096 (64)
Resignation	31	365 (54)	834 (49)
Move to another city	28	368 (54)	924 (54)
Move to the private sector	25	387 (57)	898 (53)
Stop practicing medicine	32	277 (41)	496 (29)
Feels overwhelmed	40	496 (73)	972 (57)
Suicidal ideation	29	91 (13)	252 (15)
Job strain scores			
Total job strain	21	−16 (−18 to −13)	−16 (−18 to −14)
Excessive demand subscale	21	10 (9 to 12)	11 (9 to 12)
Control subscale	21	14 (13 to 15)	15 (14 to 16)
Support subscale	21	12 (10 to 13)	12 (10 to 13)
Symptoms of severe burnout, No. (%)	27	317 (47)	635 (37)
Symptoms of severe burnout			
Emotional exhaustion	27	21 (13 to 29)	17 (10 to 26)
Depersonalization	27	6 (3 to 11)	5 (2 to 9)
Personal accomplishment	27	32 (24 to 37)	36 (29 to 41)

^a^ Symptoms of burnout were measured using the validated French-language version of the 22-item Maslach Burnout Inventory (Human Services version), which includes 3 subscales: emotional exhaustion (9 items), depersonalization (5 items), and personal accomplishment (8 items). Each item is scored from 0 (never) to 6 (every day). To assess job strain, the survey had a 12-item scale derived from the Job Content Questionnaire, with 3 domains: job demand, job control, and outside-the-job support. The job strain score was computed by subtracting the demand subscore from the sum of the control and social support subscores. The participants also completed several visual analog scales to evaluate unidimensional parameters, with 0 indicating best possible and 10 worst possible.

Of 2390 respondents, 952 (40%) reported symptoms of severe burnout. High emotional exhaustion (score ≥27) was reported by 622 respondents (26%), and 645 (27%) had high depersonalization (score ≥10). Symptoms of job strain (296 participants [12%]) and suicidal ideation (343 participants [14%]) were also reported. In all, 1206 participants (50%) had at least 1 of the 3 conditions (severe burnout, job strain, or suicidal ideation); 306 (13%) had 2 conditions, and 49 (2%) had all 3. Of the 952 participants with severe burnout, 152 (16%) had symptoms of job strain and 239 (25%) reported suicidal ideation (eFigure 1 in Supplement 1).

**Figure 1.**
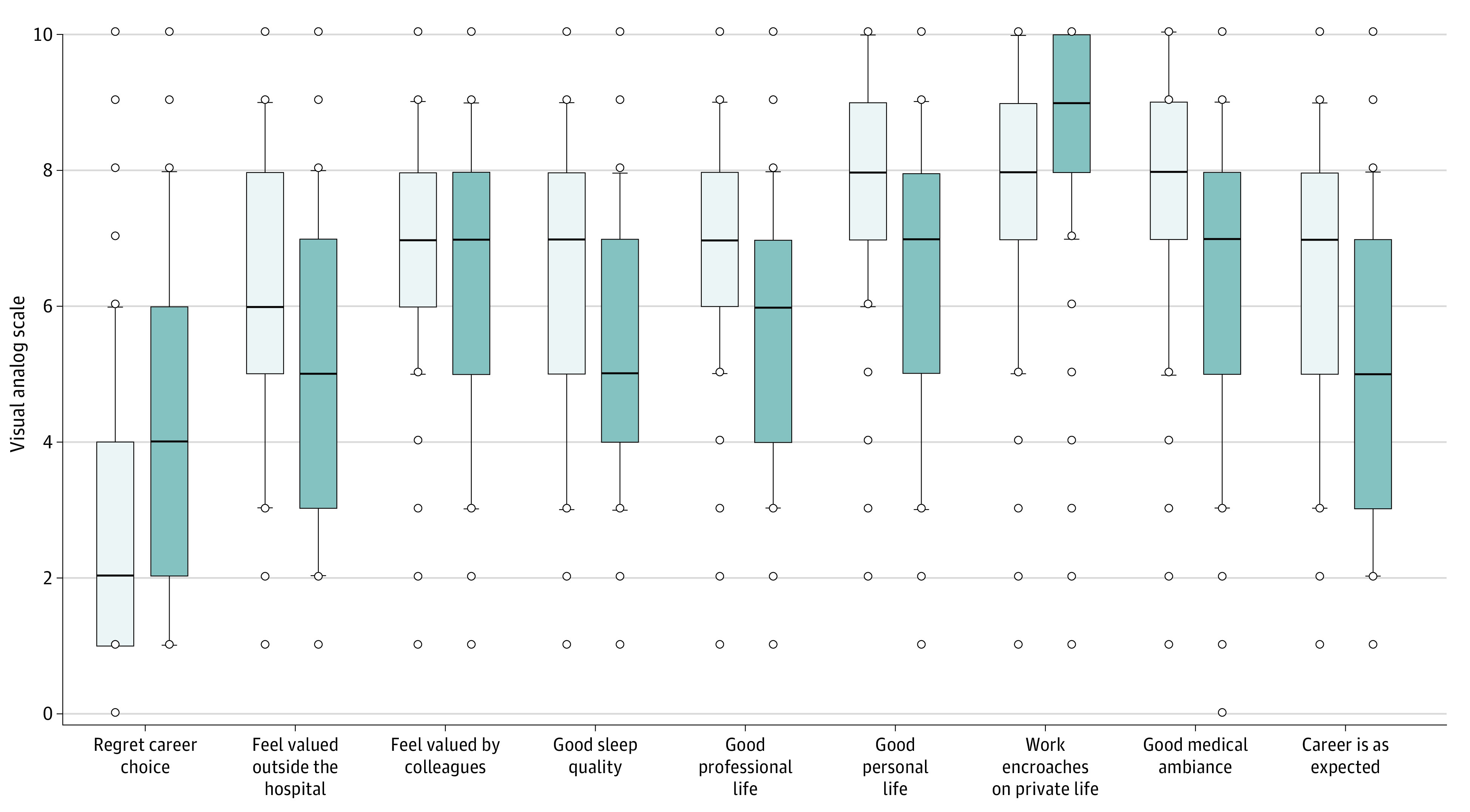
Distribution of the Visual Analog Scale Scores Used to Assess the Intensity of Unidimensional Parameters in Tenured University Hospital Professors With and Without Symptoms of Severe Burnout The scales ranged from 0 (no symptoms [best possible rating]) to 10 (most intense symptoms [worst rating]). Participants with symptoms of severe burnout are shown in dark blue and participants without symptoms in light blue. *P* < .001 for all comparisons.

As shown in eFigure 2 in Supplement 1, the following variables were independently associated with fewer symptoms of severe burnout: longer time being a professor (aOR, 0.97; 95% CI, 0.96-0.98 per year of age), sleeping well (aOR, 0.88; 95% CI, 0.83-0.92), feeling valued by colleagues (aOR, 0.91; 95% CI, 0.86-0.95 per VAS point) or the public (aOR, 0.92; 95% CI, 0.88-0.96 per VAS point), and accepting more tasks (aOR, 0.82; 95% CI, 0.72-0.93). Variables positively and independently associated with having symptoms of severe burnout were not being a clinician (aOR, 2.48; 95% CI, 1.96-3.16), reporting that work encroached on private life (aOR, 1.17; 95% CI, 1.10-1.25), feeling obligated to constantly put on a brave face (aOR, 1.82; 95% CI, 1.32-2.52), considering a career change (aOR, 1.53; 95% CI, 1.22-1.92), and having experienced harassment (aOR, 1.52; 95% CI, 1.22-1.88). Intensity of unidimensional parameters in tenured university hospital professors with and without symptoms of severe burnout is presented in Figure 1.

**Figure 2.**
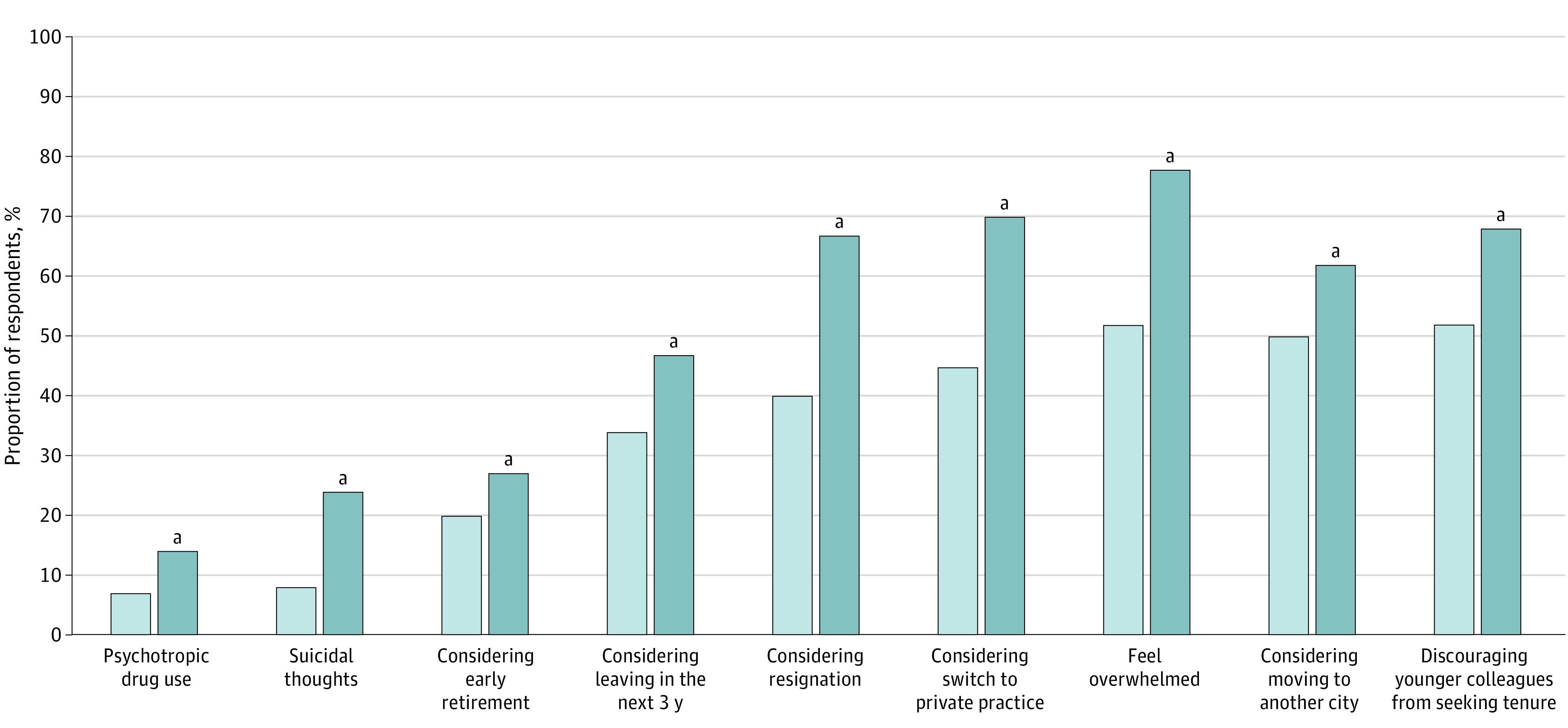
Association of the Presence of Symptoms of Severe Burnout With Professional Plans of Tenured University Hospital Professors Participants with symptoms of severe burnout are shown in dark blue and participants without symptoms in light blue. ^a^Significant difference between respondents with and without burnout (*P* < .01 for every test).

Job strain was present in 296 participants (12%). By multivariable analysis, being a long-standing professor was negatively associated with job strain (aOR, 0.98; 95% CI, 0.97-1.00 per year of age), whereas feeling that work encroached on private life showed a positive association (aOR, 1.32; 95% CI, 1.21-1.45). Presence of symptoms of severe burnout on professional plans of tenured university hospital professors is presented in Figure 2.

Suicidal ideation was reported by 343 participants (14%) and was independently associated with having a chronic illness (OR, 1.97; 95% CI, 1.47-2.62), having experienced harassment (OR, 1.89; 95% CI, 1.47-2.44), and being unable to discuss professional difficulties with colleagues (OR, 2.68; 95% CI, 1.73-4.12). Variables independently associated with not having suicidal ideation were good sleep quality (OR, 0.89; 95% CI, 0.84-0.94), ability to choose dates of days off (OR, 0.52; 95% CI, 0.32-0.82), and feeling valued by colleagues (OR, 0.93; 95% CI, 0.88-0.99) and family (OR, 0.88; 95% CI, 0.82-0.94). The final results were unchanged when men, full professors, or nonsurgeons were compared with the overall population.

## Discussion

In this cross-sectional survey of tenured university hospital faculty in France, 40% of participants had severe burnout, 12% had job strain, and 14% had suicidal ideation. Risk factors amenable to improvement were encroachment of work on private life, poor sleep quality, fraught relationships with colleagues, not regularly engaging in sports, being unable to voice concerns to colleagues, and perceived lack of support for a career change.

In France, tenured associate and full medical professors assume 3 essential missions (care, research, and teaching) and 1 transversal mission within hospitals and universities. Their status was created in 1958 to integrate care, teaching, and research into a single system. These teacher-researchers, whose main employer is the university, can teach and carry out part of their research at the hospital where they practice.

Our survey highlights the heavy workload and considerable stress imposed on university hospital professors. The workweek was long, most participants reported encroachment of work on their private life, research funding was difficult to obtain, and resources for patient care were often considered insufficient. The main encouraging findings were that most participants appreciated the climate at work, felt valued in their profession, and had control over the dates of their days off.

Symptoms of severe burnout were present in half the participants, with a higher proportion among associate than full professors. Several factors have been reported to decrease the risk of burnout, including stronger professional self-concept, support with the administrative facets of conducting research, a good balance between work and private life, and spending sufficient time on the most rewarding activities.^12,22,23^ In our survey, the participants had a preference for patient care, teaching, and research over organizational and administrative work duties, for which they felt support was insufficient. Symptoms of severe burnout were less common in participants who were older, had more experience, and were full professors, in keeping with previous studies.^24,25^ In a systematic review and meta-analysis^26^ of 15 randomized clinical trials of individual or organizational interventions (716 physicians) and 37 cohort studies (2914 physicians), the prevalence of burnout as assessed using the Maslach Burnout Inventory decreased from 54% to 44% in the intervention groups. In 1 trial, for instance, 3 monthly dinner meetings for physicians, nurses, and midwives decreased burnout and improved engagement, sense of connection to colleagues, and perceived department commitment to staff well-being.^27^ Good relationships with colleagues, meaningful interactions with learners, and protected time for personal life were crucial for full-time university hospital faculty.^28^ Facilitated small-group curriculum reduced depersonalization.^29^

In a qualitative study,^30^ factors that protected against burnout pertained to individuals (autonomy and sufficient time for nonclinical projects), teams (good adaptability, clear boundaries, and a high level of cohesion), and institutions (diversified performance evaluations and acknowledgment of individual contributions). Grit, defined as the drive to pursue long-term goals despite setbacks, was somewhat protective against burnout in a study of orthopedic surgeons.^31^ Poor sleep has often been reported to be associated with burnout.^32^ Thus, of 456 faculty radiologists, including 37.4% with burnout, 45.3% slept poorly, and this symptom correlated with burnout.^33^

Our survey also showed a high prevalence of suicidal ideation in university hospital faculty staff. Suicidality has received far more research attention in medical students than in faculty. Several risk factors were common to suicidal ideation and burnout: positive associations were found with harassment and negative associations with good-quality sleep and feeling valued by colleagues and family. Having a chronic illness was associated with suicidal ideation, as reported previously.^34^ Inability to discuss professional difficulties with colleagues was associated with suicidal ideation, suggesting that group meetings might have protective effects; however, this factor might also be an early marker for psychological distress manifesting as a sense of isolation. Group discussions are effective in improving faculty satisfaction and teamwork.^35^ Conceivably, they might also prevent friendly competition among colleagues from changing into noxious rivalry.

More associates regretted their career choice. This finding is of great concern. Recruiting the next generation of faculty is crucial to maintaining high-quality patient care, teaching, and research. Investigations are needed to identify the determinants of career choices and the factors associated with career satisfaction among university hospital faculty. Improving job attractivity is an urgent priority for university hospitals. A survey of US surgeons found that only half the participants would encourage their children to become surgeons or physicians in other specialties.^36^ The current attention to developing resiliency programs for medical students is encouraging. In a study^37^ that focused on identification of personal strengths and coping with daily stressors, faculty members who tested the program gave high ratings for usefulness, applicability, and quality. Such programs should be offered during the medical school curriculum.

### Limitations

This study has several limitations. First, all participants worked in French hospitals, and baseline mental health symptoms and perceptions of the work environment vary across countries.^38^ Moreover, organizational and societal factors specific to each country probably contribute to faculty job satisfaction and well-being.^39^ Second, the response rate was 45%. Given the anonymous data collection, we had no information on nonrespondents. Unidentified selection bias may therefore have occurred. Nonetheless, this study is among the largest and has one of the highest response rates in the field. In addition, burnout prevalence was within the previously published range.^2,12^ Third, all data were collected by self-report, and participants may have underestimated their degree of psychological distress. The extent to which an intention to change careers, resign, or retire early might result in actual change is also unclear. However, during the last decade, many associate professors in France have left university hospitals for other positions that provided better remuneration or more free time. In addition, many promising junior physicians and researchers in France have switched to other career paths before becoming tenured. Last, this survey occurred during the COVID-19 pandemic, during which symptoms of burnout were paramount in health care professionals.^40^ However, reported burnout prevalence is in the same range as in previous reports.^12^ Moreover, tenured faculty staff less frequently had mental health symptoms than other health care professionals.^41^

## Conclusions

This cross-sectional study confirms the high prevalence of symptoms of burnout, job strain, and suicidal ideation in university hospital faculty in France. Efforts to develop faculty support services in each university hospital are clearly in order. Younger faculty members were the most severely affected. Their frequently expressed intentions to change career paths is a resounding warning signal to university hospitals regarding their future ability to hire high performers. University hospitals and the administrators who manage them must tackle the current sharp downward spiral in job attractiveness in their institutions. In addition, strategies to enhance the promotion of mutual support, mutual respect, and work-life balance are warranted.

## Notes

Supplement 1. **eFigure 1.** Venn Diagram Illustrating the Overlapping of Symptoms of Severe Burnout, Suicidal Ideation, and Job Strain **eFigure 2.** Factors Independently Associated With Symptoms of Severe Burnout by Multivariable Analysis

Supplement 2.
Data Sharing Statement
