# Supplementary material for: Job Strain, Burnout, and Suicidal Ideation in Tenured University Hospital Faculty Staff in France in 2021
Source: JAMA Netw Open. 2023 Mar 28;6(3):e233652. doi: 10.1001/jamanetworkopen.2023.3652 (PMC10051074; doi:10.1001/jamanetworkopen.2023.3652)
Supplement: Supplement 1. — eFigure 1. Venn Diagram Illustrating the Overlapping of Symptoms of Severe Burnout, Suicidal Ideation, and Job Strain eFigure 2. Factors Independently Associated With Symptoms of Severe Burnout by Multivariable Analysis [file jamanetwopen-e233652-s001.pdf]

Supplementary Online Content

Dres M, Copin MC, Cariou A, et al. Job strain, burnout, and suicidal ideation in tenured university hospital faculty staff in France in 2021. *JAMA Netw Open*. 2023;6(3):e233652. doi:10.1001/jamanetworkopen.2023.3652

eFigure 1. Venn Diagram Illustrating the Overlapping of Symptoms of Severe Burnout, Suicidal Ideation, and Job Strain

eFigure 2. Factors Independently Associated With Symptoms of Severe Burnout by Multivariable Analysis

This supplementary material has been provided by the authors to give readers additional information about their work.

© 2023 Dres M et al. *JAMA Network Open.*

eFigure 1. Venn Diagram Illustrating the Overlapping of Symptoms of Severe Burnout, Suicidal Ideation, and Job Strain

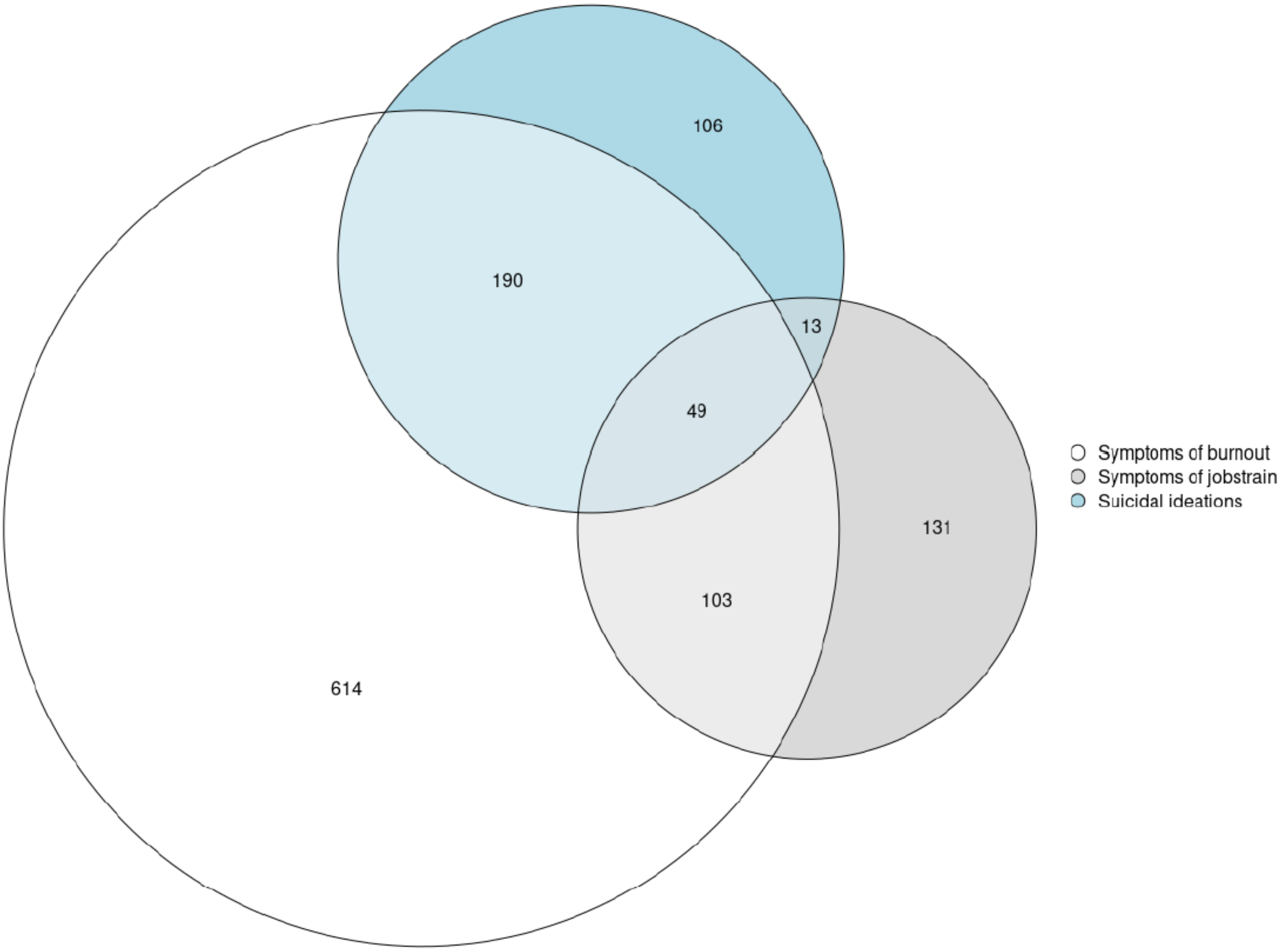

© 2023 Dres M et al. *JAMA Network Open.*

eFigure 2. Factors Independently Associated With Symptoms of Severe Burnout by Multivariable Analysis

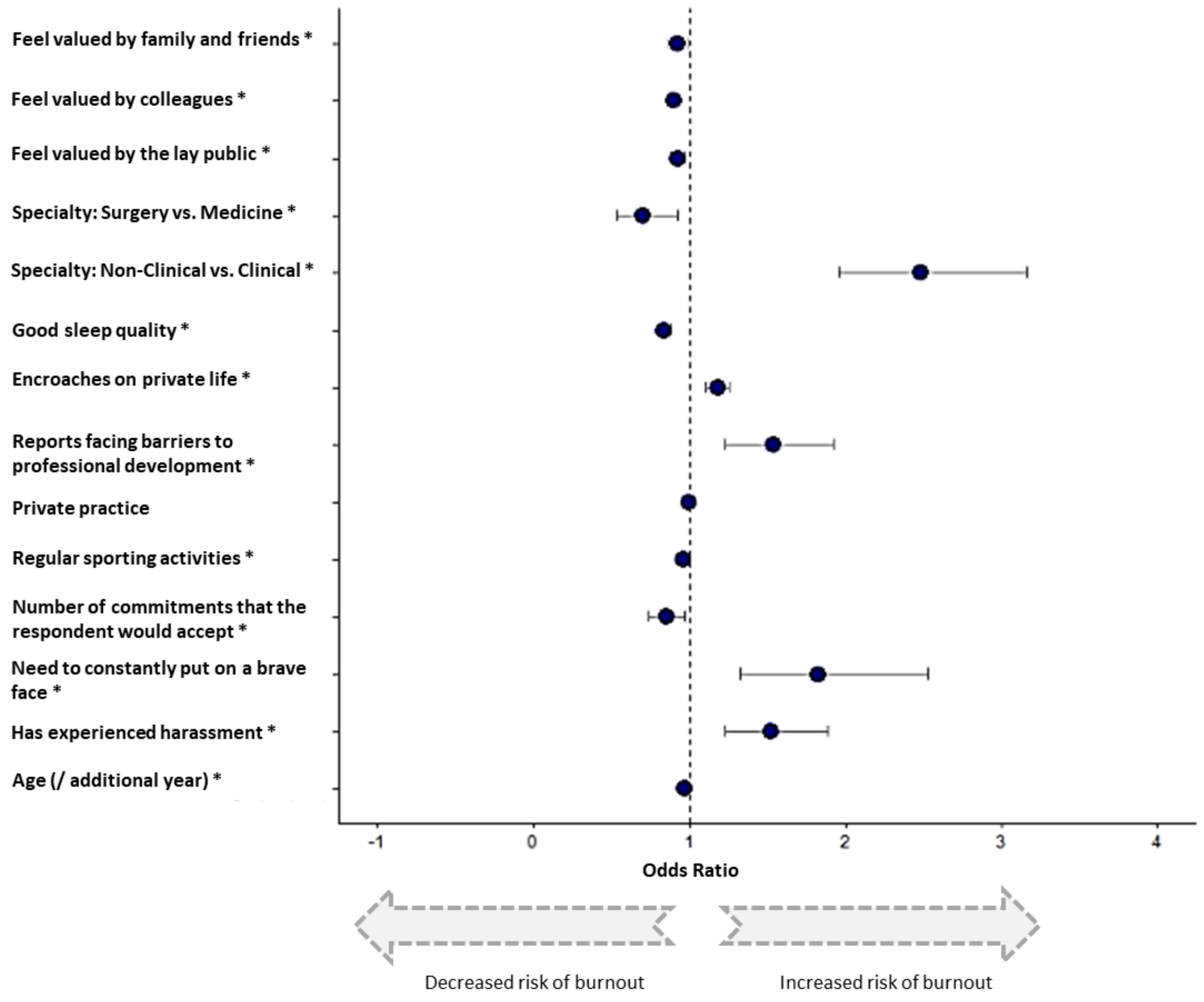

© 2023 Dres M et al. *JAMA Network Open.*
