# Supplementary material for: Job Strain, Burnout, and Suicidal Ideation in Tenured University Hospital Faculty Staff in France in 2021
Source: JAMA Netw Open. 2023 Mar 28;6(3):e233652. doi: 10.1001/jamanetworkopen.2023.3652 (PMC10051074; doi:10.1001/jamanetworkopen.2023.3652)
Supplement: Supplement 2. — Data Sharing Statement [file jamanetwopen-e233652-s002.pdf]

Data Sharing Statement

Dres. Job Strain, Burnout, and Suicidal Ideation in Tenured University Hospital Faculty Staff in France in 2021. *JAMA Netw Open*. Published March 28, 2023.
doi:10.1001/jamanetworkopen.2023.3652

Data
Data available: Yes
Data types: Deidentified participant data
How to access data: Access to the data by asking the corresponding author
When available: With publication

Supporting Documents
Document types: None

Additional Information
Who can access the data: researchers
Types of analyses: systematic reviews
Mechanisms of data availability: signed data access agreement
Any additional restrictions: none
